# Innovative Use of *Spirogyra* sp. Biomass for the Sustainable Adsorption of Aflatoxin B_1_ and Ochratoxin A in Aqueous Solutions

**DOI:** 10.3390/molecules29215038

**Published:** 2024-10-25

**Authors:** Wipada Siri-anusornsak, Oluwatobi Kolawole, Siriwan Soiklom, Krittaya Petchpoung, Kannika Keawkim, Chananya Chuaysrinule, Thanapoom Maneeboon

**Affiliations:** 1 Scientific Equipment and Research Division, Kasetsart University Research and Development Institute (KURDI), Kasetsart University, Bangkok 10900, Thailand; rdisws@ku.ac.th (S.S.); rdikyp@ku.ac.th (K.P.); rdicnc@ku.ac.th (C.C.); rditpm@ku.ac.th (T.M.); 2 Plant Sciences Group, Wageningen University & Research, Droevendaalsesteeg 4, 6708 PB Wageningen, The Netherlands; tobi.kolawole@wur.nl; 3 Division of Physical Science, Faculty of Science and Technology, Huachiew Chalermprakiet University, Bang Chalong, Samut Prakan 10540, Thailand; k.kannika2252@gmail.com

**Keywords:** adsorption, adsorbents, algal biomass, mitigation, mycotoxin removal, *Spirogyra* sp.

## Abstract

This research investigates the efficacy of *Spirogyra* sp. biomass as an effective adsorbent for the removal of AFB_1_ and OTA from aqueous solutions. Several factors, including contact time, adsorbent dosage, pH level, and initial mycotoxin concentration, were analyzed to evaluate their impact on adsorption efficacy. The optimal contact time for equilibrium was determined at 60 min, during which the TPA obtained a 91% reduction in AFB_1_ and 68% removal of OTA. Although increasing the adsorbent dosage improved effectiveness, excessive quantities led to particle aggregation, hence diminishing adsorption performance. The optimal dosage of 5.0 mg/mL optimized the efficacy and use of resources. Adsorption was more efficacious at acidic to neutral pH levels (5–6), enhancing the accessibility of functional groups on the biomass. Kinetic analysis indicated that adsorption process followed a pseudo second-order model, whereas isotherm studies demonstrated a heterogeneous adsorption mechanism, with the Freundlich model providing the optimal fit. The TPB exhibited enhanced adsorption capacities for both mycotoxins, offering a viable solution for mitigating mycotoxin contamination in food and feed. These findings illustrate the significance of biomass treatment techniques in improving mycotoxin removal efficacy and suggest the potential of algal biomass in food safety applications.

## 1. Introduction

Mycotoxins are toxic secondary metabolites produced by fungi that contaminate food and feed, posing significant threats to both human and animal health. Among the diverse range of mycotoxins, aflatoxin B_1_ (AFB_1_) and ochratoxin A (OTA) are of particular concern due to their potent toxicity and widespread occurrence in agricultural commodities [1,2,3]. AFB_1_, produced by *Aspergillus* species, is classified as a Group 1 carcinogen by the International Agency for Research on Cancer (IARC) due to its strong association with liver cancer and other severe health issues, which has prompted stringent regulatory limits on its presence in food and feed [4]. OTA, primarily produced by *Aspergillus* and *Penicillium* species, is known for its nephrotoxic, hepatotoxic, and immunotoxic effects, making it a critical food safety concern [5,6]. The contamination of food and feed with AFB_1_ and OTA is a global challenge with far-reaching consequences. These mycotoxins can persist throughout the food chain, leading to acute and chronic health problems in humans and animals [6,7]. To mitigate these risks, numerous countries have implemented strict regulations to control mycotoxin levels in food and feed products [8].

Current methods for the removal of mycotoxins, including physical, chemical, and biological approaches, face challenges in terms of efficiency, cost-effectiveness, and potential negative impacts on food quality [9,10]. For instance, physical methods such as sorting and washing are often inadequate for complete removal, while chemical treatments may leave harmful residues. Biological approaches, including the use of enzymes or microorganisms, hold promise but require further development for practical applications.

In this context, adsorption technology has gained increasing attention as an efficient and environmentally friendly method for mycotoxin removal. Adsorption processes involve the binding of contaminants to a solid adsorbent material, which can be used to purify aqueous systems. Among various adsorbents, natural biomass materials have emerged as sustainable alternatives due to their low cost, availability, and environmental compatibility [11]. Algal biomass, in particular, has demonstrated potential in adsorbing various pollutants, including heavy metals and organic contaminants, due to its complex cell wall structure, which is rich in functional groups like hydroxyl, carboxyl, and amino groups [12].

*Spirogyra* sp., a filamentous green alga, has attracted interest as a potential biosorbent due to its abundance and eco-friendliness [13]. Its biomass, often regarded as a byproduct of bioactive compound extraction processes, is highly porous and contains surface functional groups that can interact with mycotoxins through mechanisms such as hydrogen bonding and electrostatic interactions. The use of *Spirogyra* sp. biomass aligns with the principles of green chemistry, offering a sustainable solution for reducing mycotoxin contamination in water and food systems.

Despite its potential, there remains a need for comprehensive studies evaluating the use of *Spirogyra* sp. biomass for the adsorption of AFB_1_ and OTA in aqueous environments. Understanding the factors that affect adsorption efficiency, including pH, contact time, and adsorbent dosage, is crucial for optimizing the process and expanding its practical applications.

This study aims to assess the adsorption capacity of *Spirogyra* sp. biomass for the removal of AFB_1_ and OTA from aqueous solutions. By systematically investigating the influence of key operational parameters and exploring the underlying adsorption mechanisms, this research seeks to provide valuable insights into the development of sustainable biosorbents for mycotoxin decontamination. The findings will contribute to the advancement of environmentally friendly strategies for ensuring food and feed safety.

## 2. Results and Discussion

### 2.1. Adsorbent Properties and Evaluation

#### 2.1.1. SEM Images

**Figure 1:**
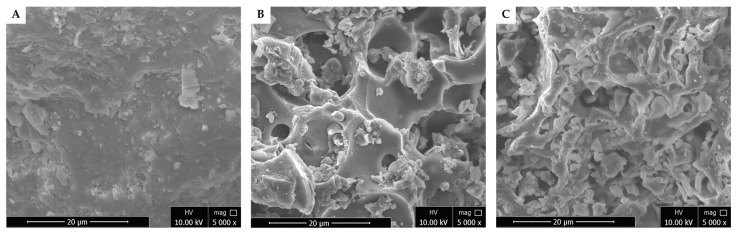
SEM images of algal powder (**A**), acid−treated algal powder (**B**), and alkali−treated algal powder (**C**) at ×5000 magnification and 10 kV.

Scanning electron microscopy (SEM) was used to observe the surface morphology changes of TP before and after modification. The SEM analysis was conducted to characterize the surface morphology of the TP, TPA, and TPB adsorbents. The untreated TP displayed a smooth, sheet-like structure (Figure 1A). In contrast, both TPA and TPB exhibited significantly altered morphologies with rougher surfaces, larger pores, and increased void spaces (Figure 1B,C). These structural modifications are attributed to the acid and alkali treatments, which are known to enhance surface area and introduce functional groups [14]. The increased porosity and surface heterogeneity of TPA and TPB suggest a greater potential for adsorbing molecules compared to the untreated TP.

SEM analysis revealed that TP had a smooth, sheet-like structure, while the chemically modified TPA and TPB exhibited rougher surfaces, larger pores, and increased void spaces, consistent with findings in previous studies. For example, acid treatments applied to activated carbon and coking coal have been shown to increase surface roughness, promote pore development, and enhance adsorption capacity [15,16]. Similarly, alkaline treatment of biomass typically increases surface area and porosity by removing lignin and hemicellulose [17]. These structural modifications improve the ability of adsorbents to interact with target molecules, such as AFB_1_ and OTA. The SEM results clearly illustrate that chemical treatments effectively enhance the adsorptive properties of algal biomass. The resulting increases in surface area and pore volume are key factors contributing to the improved adsorption capacity of the materials.

#### 2.1.2. BET Surface Area

**Figure 2:**
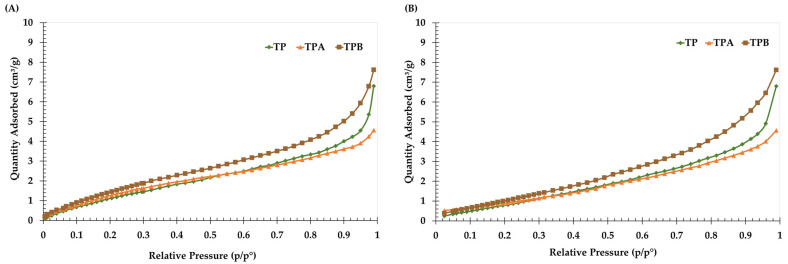
N_2_ adsorption–desorption isotherms (**A**,**B**) of algal powder (TP); acid−treated algal powder (TPA); alkali−treated algal powder (TPB).

Nitrogen (N_2_) adsorption–desorption isotherms for TP (untreated), TPA (acid-treated), and TPB (alkali-treated) samples (Figure 2) exhibit Type IV isotherms, as classified by BDDT [18], confirming mesoporosity in all samples. A slight hysteresis in the desorption branch suggests a variation in pore size distribution within the materials. Pore volume, pore size distribution, and surface area are the main parameters affecting activated carbons’ adsorption performance [19].

The porous structure characteristics, including BET surface area and pore size distribution, were determined using the BJH method, with the results presented in Table 1. Although the TPA and TPB samples display minor increases in surface area and slight reductions in pore size compared to TP, these modest changes alone do not appear sufficient to account for the enhanced adsorption performance.

The BET analysis indicates that TP, with a surface area of 6.39 m^2^/g and an average pore size of 75.93 Å, qualifies as a mesoporous material under IUPAC standards. The chemically treated TPA and TPB samples, although slightly improved in surface area and reduced in average pore size, maintain a mesoporous structure. Notably, the pore volume of TPB remains similar to that of TP, indicating that sodium hydroxide treatment has limited impact on pore volume, though some improvement in surface area and textural characteristics is observed. NaOH treatment promotes the formation of surface hydroxyl groups, increasing surface basicity by introducing electron-donating sites. This process deprotonates acidic functional groups, reducing surface acidity and creating negatively charged sites that enhance the adsorbent’s affinity for positively charged species, such as metal ions or protonated organic molecules. The treatment may also enlarge pores, improving access to binding sites and potentially enhancing adsorption capacity [20].

The TPA showed a BET surface area of 6.77 m^2^/g, representing a minor increase from the TP, which had a surface area of 6.39 m^2^/g. The sulfuric acid (H_2_SO_4_) is beneficial for enhancing surface area and total volume. A review of the impact of chemical modification indicated that acid treatment produces a positive surface charge, hence enhancing the adsorption of positively charged metal ions [14]. Treatment with sulfuric acid often enhances pore development, resulting in an increased surface area. This process indicates that sulfuric acid may encourage the formation of more active sites and simultaneously create an electrical double layer on the adsorbent’s surface. These modifications enhance the adsorbent’s overall adsorption capability by increasing the availability of reactive sites for interaction with target contaminants [14]. Although this increase in surface area and porosity is slight, it does not fully account for the significant improvement in adsorption performance observed with TPA.

The N_2_ adsorption–desorption isotherms confirmed that TP, TPA, and TPB exhibit mesoporous characteristics. Although the changes in BET surface area were relatively small, these modifications can influence adsorption through mechanisms beyond mere pore structure. The acid and alkali treatments likely enhanced the binding affinity of adsorbent for mycotoxins by creating new active sites. Specifically, these treatments may have led to the formation of additional pores and minor enlargements in existing ones, improving accessibility to these active sites within the adsorbent matrix. The enhanced surface area and porosity of the treated samples, as evidenced by BET analysis, correlate well with morphological observations from SEM. The increased surface area in TPA and TPB can be attributed to the formation of new pores and the expansion of existing ones during chemical treatment. Notably, the reduced average pore size in TPA suggests the formation of smaller pores, which may improve the accessibility of mycotoxins to the adsorbent surface. The mesoporous structure was maintained across all samples, indicating that the chemical treatments did not significantly alter the overall pore size distribution. The observed correlation between increased surface area and enhanced adsorption capacity, as demonstrated in previous experiments, reinforces the importance of pore structure in the adsorption process. The larger surface areas of TPA and TPB provide additional binding sites for mycotoxins, leading to improved adsorption efficiency. Overall, these findings underscore the beneficial effects of chemical modifications on the adsorptive properties of algal biomass [21].

#### 2.1.3. FT-IR Spectroscopy

**Figure 3:**
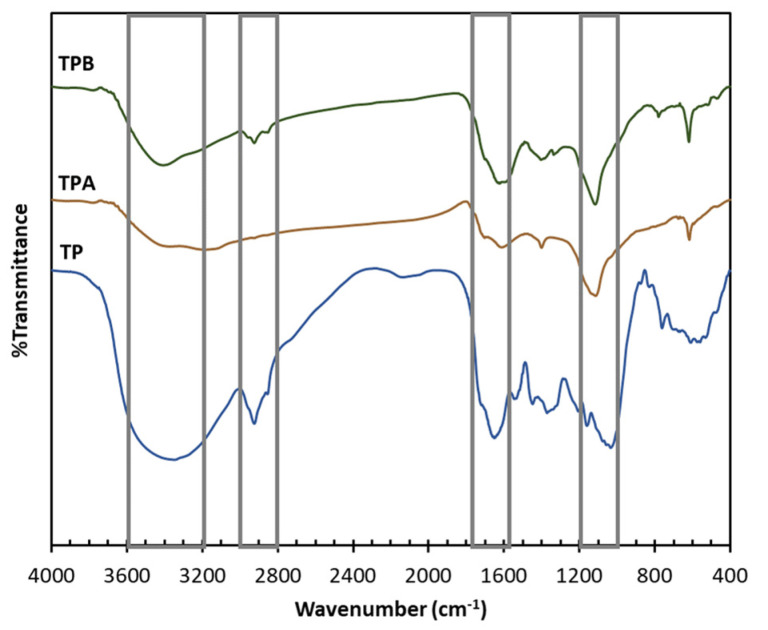
FTIR spectra of algal powder (TP); acid−treated algal powder (TPA); alkali−treated algal powder (TPB).

The FTIR spectra of the samples (Figure 3) illustrate several key functional groups present on the surface of the prepared materials. A broad band in the 3600–3100 cm^−1^ region corresponds to O-H stretching vibrations associated with cellulose hydroxyl groups [22,23]. Bands between 3000 and 2800 cm^−1^ confirm C-H stretching vibrations [24,25], and their persistence in the NaOH-treated samples suggests that the treatment process preserved the cellulose structure. The carbonyl (C=O) group, observed in the 1800–1500 cm^−1^ region, indicates the presence of functional groups, such as carboxylic and hydroxyl, which could serve as adsorption sites [26]. Additionally, the C-O stretching vibrations in the 1200–1036 cm^−1^ range, which remained unchanged by chemical treatment, reflect the stability of lignin within the samples [27]. However, the functional groups observed may benefit from further enhancement to improve adsorption performance. The presence and accessibility of specific functional groups—such as carboxylic, hydroxyl, and carbonyl groups—are essential in facilitating effective binding with target molecules, such as mycotoxins. While the current chemical treatments retain key structures, they may not significantly increase the density or reactivity of these active sites. Optimizing functional group characteristics on the surface could enhance adsorption by increasing the number and accessibility of binding sites.

The FTIR spectra of the samples provide significant insights into the functional group properties on the material’s surface. Improving the accessibility and density of these functional groups may be a critical focus for future study to enhance adsorption efficiency. Enhancing or including additional active sites can significantly enhance the material’s efficacy as an adsorbent. Future research may examine improved chemical treatments or look for other modification methods to enhance functional group density and generate additional reactive sites. Introducing or selectively augmenting specific functional groups may significantly enhance adsorption efficiency. These developments may result in more precise and efficient adsorbents, greatly enhancing the material’s capacity for sequestering mycotoxins and other contaminants.

### 2.2. Effect of Contact Time

**Figure 4:**
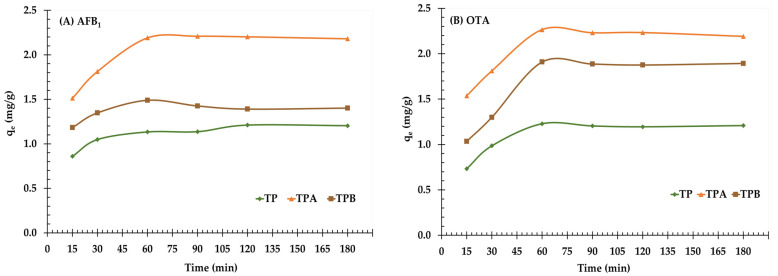
The kinetic curves for adsorption of aflatoxin B_1_ (**A**) and ochratoxin A (**B**) onto algal powder (TP); acid−treated algal powder (TPA); alkali−treated algal powder (TPB) at 15–180 min.

The adsorption capacities of AFB_1_ and OTA on TP, TPA, and TPB were investigated over a range of contact times (15–180 min). The adsorption capacities of each adsorbent were determined and are presented in Figure 4.

The adsorption for all three adsorbents showed a rapid initial increase in mycotoxin removal, which can be attributed to the abundance of available binding sites at the beginning of the process. As these binding sites became occupied, the adsorption rate decreased, eventually reaching equilibrium within 60 min for all adsorbents. At equilibrium, TPA exhibited the highest adsorption capacities, achieving 91% for AFB_1_ and 68% for OTA, confirming its efficacy in mycotoxin removal.

The adsorption capacity of TP for AFB_1_ increased from 0.86 mg/g at 15 min to a high of 1.21 mg/g at 120 min, after that exhibiting an insignificant reduction to 1.20 mg/g at 180 min. TPA exhibited the highest adsorption capacity among the investigated materials, achieving 2.21 mg/g at 60 min and maintaining a consistent level around this maximum until 180 min. TPB had a moderate adsorption capability, increasing from 1.18 mg/g at 15 min to 1.49 mg/g at 60 min, then decreasing and maintaining at around 1.40 mg/g at 180 min.

For OTA, the adsorption capacity of TP increased from 0.73 mg/g at 15 min to 1.21 mg/g at 180 min. TPA demonstrated a high adsorption capability, obtaining 2.27 mg/g at 60 min, followed by an insignificant decrease to 2.19 mg/g after 180 min. TPB exhibited a slow increase in adsorption capacity, beginning at 1.03 mg/g after 15 min and maintaining at around 1.89 mg/g after 180 min.

In both AFB_1_ and OTA, TPA consistently exhibited the highest adsorption capabilities, followed by TPB and then TP. The TPA demonstrated greater effectiveness in adsorbing both toxins, probably related to the increased surface area and improved functional group accessibility resulting from acid treatment. The adsorption capacity of each adsorbent was maintained after 60–90 min, showing that equilibrium was attained for both AFB_1_ and OTA during this period.

The contact time study revealed that TPA exhibited the highest adsorption capacity for both AFB_1_ and OTA, reaching equilibrium within 60 min. This performance can be attributed to several factors, including a larger surface area, higher pore volume, and the presence of specific functional groups capable of interacting with mycotoxins. The rapid initial adsorption phase observed for all adsorbents suggests the availability of abundant active sites on the adsorbent surface. The subsequent decrease in adsorption rate indicates the gradual saturation of these sites. The longer contact time required for equilibrium compared to other adsorbents, such as grape pomace [28] and banana peel [23] adsorbents, highlights the potential influence of adsorbent structure and pore diffusion characteristics on the adsorption kinetics. The complex pore structure of algal biomass may impede the rapid diffusion of mycotoxins within the adsorbent matrix, resulting in slower adsorption rates.

The results indicate that the adsorption capacity of TPA is attributed to its greater surface area, increased pore volume, and functional groups; nonetheless, it is crucial to understand that contact duration may interact with pH and adsorbent dosage, thereby affecting adsorption effectiveness. The ideal contact duration might vary at varying pH levels due to alterations in adsorbent surface charge or mycotoxin speciation, influencing the accessibility and binding of mycotoxins to active sites.

### 2.3. Effect of Adsorbent Dosage

**Figure 5:**
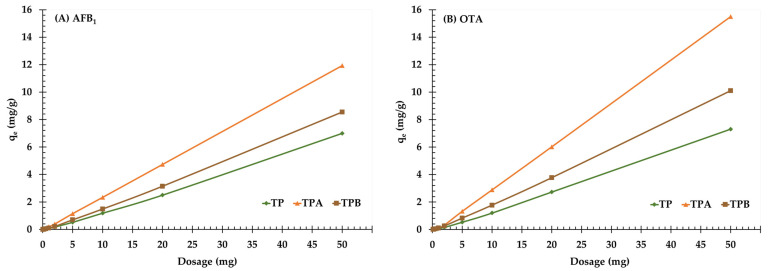
Kinetic curves for the adsorption of aflatoxin B_1_ (**A**) and ochratoxin A (**B**) onto algal powder (TP); acid−treated algal powder (TPA); alkali−treated algal powder (TPB) at various adsorbent dosages.

The impact of adsorbent dosage on AFB_1_ and OTA removal was investigated by varying the amount of adsorbent from 0.01 to 50 mg/mL while maintaining a constant mycotoxin concentration (1.0 mg/L), pH (3.0), and contact time (60 min). The results are shown in Figure 5; a positive correlation was observed between adsorbent dosage and adsorption efficiency for both mycotoxins.

The adsorption capacity for AFB_1_ was reduced as the toxin dosage decreased from 50 mg to 0.01 mg for all adsorbents. TP demonstrated an adsorption capacity of 6.99 mg/g at a dosage of 50 mg, which significantly decreased to 2.50 mg/g and 1.18 mg/g at dosages of 20 mg and 10 mg, respectively. At the smallest dosage of 0.01 mg, TP exhibited negligible adsorption (0.0006 mg/g). This trend indicates that TP reduced binding sites and had comparatively lower affinity for AFB_1_ than TPA and TPB. The TPA consistently had the greatest adsorption capacity at all doses. The adsorption capacity started at 11.93 mg/g for a 50 mg dosage, subsequently decreasing to 4.73 mg/g at 20 mg, 2.34 mg/g at 10 mg, and lastly reducing to 0.0006 mg/g at the minimum dosage of 0.01 mg. The TPA revealed better performance, indicating that acid treatment improved its surface characteristics, resulting in increased active binding sites for AFB_1_, even at decreased toxin concentrations. TPB exhibited intermediate adsorption capabilities relative to TP and TPA. At a dosage of 50 mg, TPB adsorbed 8.55 mg/g of AFB_1_, which decreased to 3.15 mg/g at 20 mg and 1.49 mg/g at 10 mg. The adsorption capacity further decreased to 0.0005 mg/g at the 0.01 mg dosage level. The results indicate that alkali treatment considerably enhanced the affinity of adsorbent for AFB_1_, though it was less efficient than acid treatment.

For OTA, TP showed an initial adsorption capacity of 7.31 mg/g at a dosage of 50 mg, which decreased to 2.73 mg/g at 20 mg, 1.20 mg/g at 10 mg, and lastly 0.0034 mg/g at the 0.1 mg dosage level. This decrease indicates the lower surface area and binding affinity of TP relative to the treated adsorbents. TPA demonstrated the greatest adsorption capability for OTA at all doses, similar to its efficacy with AFB_1_. At a dosage of 50 mg, TPA obtained an adsorption capacity of 15.50 mg/g, which was reduced to 6.02 mg/g at 20 mg and 2.89 mg/g at 10 mg. At lower concentrations, specifically 0.1 mg, TPA had a notable adsorption capacity of 0.0041 mg/g, underscoring its effective binding sites and enhanced interactions resulting from acid treatment. TPB revealed intermediate adsorption values, with a capacity of 10.10 mg/g at a dosage of 50 mg, 3.77 mg/g at 20 mg, and 1.77 mg/g at 10 mg. At a dosage of 0.1 mg, the adsorption capability of TPB decreased to 0.0050 mg/g. This performance indicates that alkali treatment moderately improved OTA adsorption relative to TP, while it was less efficient than TPA.

TPA demonstrated the highest adsorption capacities for both AFB_1_ and OTA across all dosages, emphasizing the efficacy of acid treatment in enhancing adsorbent binding characteristics. TPB, though improved by alkali treatment, was less efficient than TPA but more effective than TP, highlighting the importance of surface modifications in optimizing adsorbent performance for mycotoxin removal.

The adsorption capacities of TP, TPA, and TPB for AFB_1_ and OTA indicate significant differences in adsorbent effectiveness, with TPA consistently showing the highest capabilities at all dose levels. This trend emphasizes that TPA improved efficacy, probably because of enhanced surface properties from acid treatment, which may have improved the quantity and accessibility of active binding sites.

The findings indicate that enhancing the adsorbent dosage initially enhances mycotoxin removal efficacy, but only to a specific level; further increases in dosage result in negligible improvements. Excessive amounts of adsorbent might result in particle aggregation, decreasing the effective surface area and hence limiting adsorption performance [29,30]. The increased effectiveness of TPA can likely be attributed to its unusual physicochemical characteristics, including an extensive surface area, optimum pore structure, and the presence of functional groups that enhance interactions with mycotoxins. Additional research will be required to clarify the particular mechanisms leading to the enhanced adsorption capacity of TPA. The optimal dosage of 5.0 mg/mL determined in this study achieves a balance between enhancing adsorption efficiency and reducing the quantity of adsorbent utilized. Several criteria must be considered when establishing the optimal dosage for practical applications, including the initial mycotoxin concentration, the intended decrease level, and the cost-effectiveness of the adsorption procedure.

Furthermore, it is essential to examine the interaction among adsorbent dose, starting mycotoxin concentration, and pH. Although increased dosages might mitigate the impact of increased initial concentrations, the efficacy of this approach is dependent upon pH conditions, as variations in pH can affect surface charge and the availability of binding sites. Insufficient doses of adsorbent may result in particle aggregation, hence reducing the effective surface area and ultimately decreasing adsorption efficiency.

### 2.4. Effect of pH Solution

**Figure 6:**
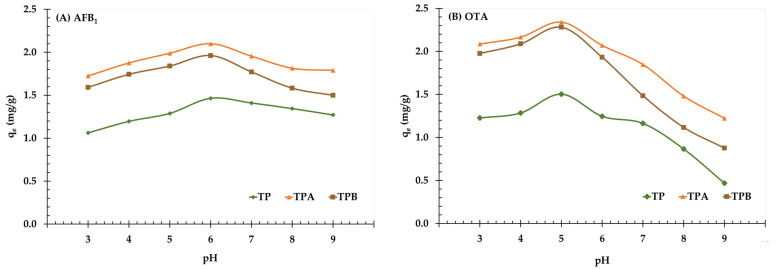
The kinetic curves for adsorption of aflatoxin B_1_ (**A**) and ochratoxin A (**B**) onto algal powder (TP); acid−treated algal powder (TPA); alkali−treated algal powder (TPB) at pH 3–9.

The pH significantly influences adsorption processes by affecting both adsorbent surface charge and mycotoxin speciation. The adsorption efficiency of TP, TPA, and TPB for AFB_1_ and OTA was evaluated across a pH range of 3 to 9 (Figure 6).

For AFB_1_, TPA showed the greatest adsorption capacities across all pH levels, achieving a maximum of 2.0997 mg/g at pH 6. This was followed by TPB, which had a maximum capacity of 1.9620 mg/g at pH 6, and TP, which reached 1.4645 mg/g at pH 6. This indicates that TPA demonstrates a greater affinity for AFB_1_ at different pH levels, with its adsorption capacity decreasing slightly at high pH levels (pH 7–9). Similar trends have been observed for OTA. TPA displayed the greatest adsorption capability across all pH levels, obtaining an optimal level of 2.3399 mg/g at pH 5. TPB showed intermediate adsorption capacities, reaching at 2.2809 mg/g at pH 5, whereas TP displayed the lowest capacities, obtaining a high of 1.5029 mg/g at pH 5. Significantly, OTA adsorption decreased at high pH levels (pH 8–9) across all adsorbents, especially for TP, which exhibited a pronounced reduction.

The findings indicate that acidic to neutral pH conditions (pH 5–6) enhance the adsorption of both AFB1 and OTA across all adsorbents, especially TPA, which consistently exceeded TP and TPB in performance. The increased adsorption capacity of TPA is likely attributable to the acid treatment, which may enhance the surface characteristics and functional groups, hence improving the interaction with mycotoxins at different pH levels. Additional research is required to examine the impact of pH on the properties of functional groups and the surface charge of the adsorbents.

The results of this investigation reveal significant differences in the adsorption capacities of the three adsorbents—TP, TPA, and TPB—regarding AFB_1_ and OTA across different pH levels. TPA exhibited high adsorption capacity for both mycotoxins across a broad pH range, highlighting its versatility as an adsorbent. The optimal pH for AFB_1_ adsorption was found to be 6, while the highest adsorption efficiency for OTA was observed at pH 5. These findings suggest that TPA possesses favorable surface properties and functional groups that interact effectively with mycotoxins under varying pH conditions.

In contrast, TP showed lower adsorption efficiency, particularly at alkaline pH, likely due to its surface chemistry and pore structure. The reduced availability of binding sites or competition from hydroxyl ions at higher pH may contribute to TP’s decreased performance. Previous studies have emphasized the importance of surface area and pore structure in adsorption processes [29]. The enhanced surface properties of TPA and TPB compared to TP likely contribute to their superior performance. Additionally, adsorbent density and competition for binding sites can significantly influence adsorption efficiency [23].

Therefore, TPA is identified as the optimal adsorbent for removing AFB_1_ and OTA from aqueous solutions, especially within a neutral pH range. However, specific pH conditions should be tailored based on the target mycotoxin and desired adsorption efficiency. It is also essential to consider the interactions between pH, contact time, and adsorbent dosage. For example, varying pH levels can alter binding site accessibility and surface charge density, necessitating adjustments in contact time or adsorbent dosage to achieve optimal adsorption. The reduced performance of TP at higher pH values may be linked to competition from hydroxyl ions for adsorption sites, decreasing its efficacy in alkaline conditions.

### 2.5. Effect of the Initial Mycotoxin Concentration

**Figure 7:**
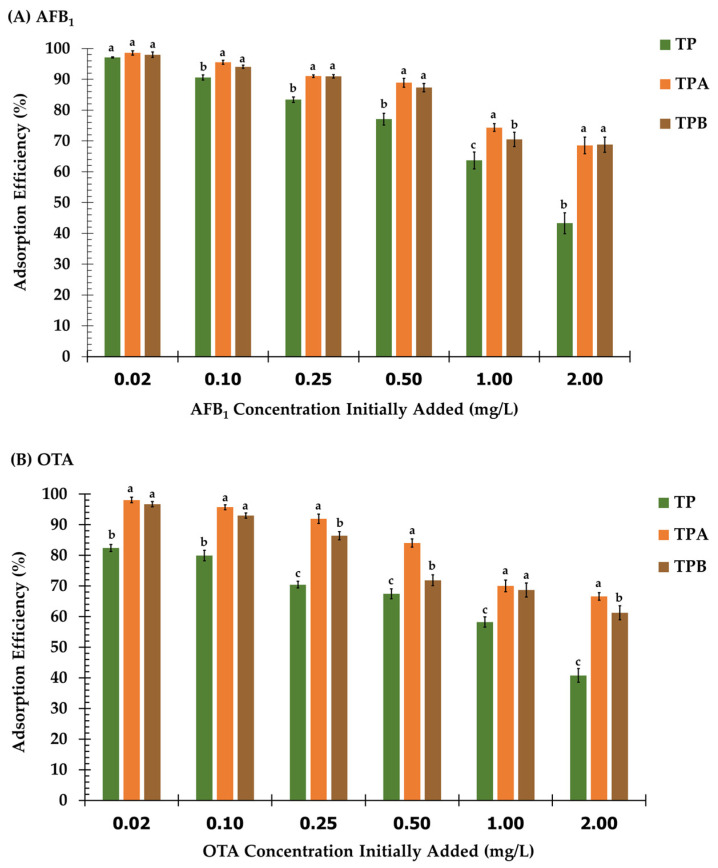
Adsorption efficiency of algal powder (TP); acid−treated algal powder (TPA); alkali−treated algal powder (TPB) at different concentrations of (**A**) aflatoxin B_1_ and (**B**) ochratoxin A. ^a,b,c^ mean different letters in the same group indicate significant differences (*p* < 0.05).

The adsorption capacities of TP, TPA, and TPB for AFB_1_ and OTA were evaluated across a concentration range of 0.02 to 2.0 mg/L (Figure 7). At the lowest concentration (0.02 mg/L), all adsorbents exhibited high removal efficiencies, with TPA and TPB demonstrating high performance for both mycotoxins compared to TP.

Figure 7A illustrates the adsorption efficiency of AFB1 across various concentrations for each adsorbent. TPA showed enhanced adsorption efficiency at all investigated concentrations in comparison to TP and TPB. At a concentration of 0.02 mg/L, TPA obtained an adsorption efficiency of 98.56%, while TP and TPB had efficiencies of 97.15% and 97.98%, respectively. At a concentration of 2.00 mg/L, TPA revealed an efficiency of 68.53%, whereas TP showed an efficiency of 43.30% and TPB achieved an efficiency of 68.81%. The adsorption efficiencies of OTA at different concentrations are presented in Figure 7B. TPA demonstrated the highest adsorption efficiencies at all dosages, achieving 98.08% at 0.02 mg/L and 66.58% at 2.00 mg/L. The performance of TP showed a significant decrease, achieving an optimal efficiency of 82.42% at 0.02 mg/L, which reduced to 40.78% at 2.00 mg/L. TPB exhibited intermediate efficiency, achieving 96.71% at 0.02 mg/L and 61.23% at 2.00 mg/L.

As the initial mycotoxin concentration increased, adsorption efficiencies gradually declined for all adsorbents. However, TPA consistently maintained high removal rates for both AFB_1_ and OTA across the entire concentration range. TPB exhibited intermediate performance, while TP demonstrated the lowest adsorption capacities, particularly at higher concentrations. The observed trend can be attributed to the limited availability of adsorption sites on the adsorbents at higher mycotoxin concentrations. TPA and TPB, with their potentially larger surface areas and more abundant binding sites, exhibited greater capacity to accommodate increasing mycotoxin loads compared to TP.

The findings of this investigation indicate that TPA displays enhanced adsorption efficacy for both AFB_1_ and OTA throughout a broad spectrum of concentrations in comparison to the other adsorbents, TP and TPB. The improved performance highlights the importance of chemical modifications applied to TPA via acid treatment, which probably enabled the addition of reactive functional groups, thus improving binding interactions with mycotoxins.

The interaction between initial mycotoxin concentration and adsorbent dosage is crucial for real-world applications. Higher concentrations may require increased adsorbent dosages to maintain efficiency. Additionally, this interaction can be influenced by pH, as certain pH levels may promote adsorbent agglomeration, further reducing effective surface area and adsorption efficiency. The study examines the individual effects of pH, contact time, adsorbent dosage, and mycotoxin content; however, interactions among these variables are expected to significantly influence the optimization of adsorption performance. Future research may employ a factorial design to thoroughly examine these connections. This would provide a more accurate assessment of optimum conditions, guaranteeing effective mycotoxin elimination across diverse environmental conditions and mycotoxin concentrations.

### 2.6. Mycotoxin Adsorption and Desorption Study

The objective of this study was to explore the interactions between mycotoxins and different adsorbents, and to assess the conditions under which mycotoxins might be released or desorbed from these adsorbents. Understanding these dynamics is crucial for evaluating the stability and effectiveness of mycotoxin mitigation strategies. To assess the potential for toxin release due to pH shifts in the environment, a desorption experiment was performed following the methodology of Greco et al. [30].

**Figure 8:**
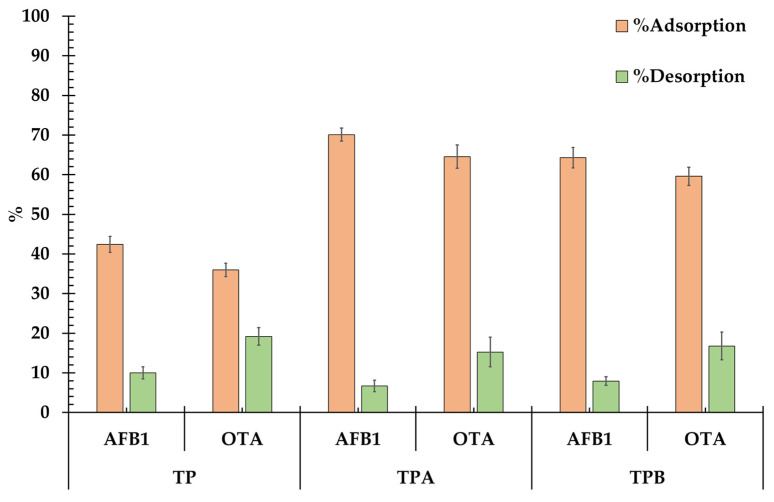
Adsorption and desorption characteristics of AFB_1_ and OTA onto TP, TPA, and TPB.

The adsorption and desorption characteristics of AFB_1_ and OTA onto TP, TPA, and TPB are summarized in Figure 8. Adsorption was tested at pH 3, simulating conditions found in the stomach, while desorption was evaluated at pH 7, representing conditions in the small intestine.

Significant differences in adsorption capacities were observed across the three samples. TP exhibited the lowest adsorption for both AFB_1_ (42.42%) and OTA (35.96%), with statistically lower values (*p* < 0.05) compared to the treated samples. In contrast, TPA demonstrated the highest adsorption, with 70.09% for AFB_1_ and 64.54% for OTA. This suggests that acid treatment significantly enhances the adsorption capability of algal biomass. TPB also showed improved adsorption relative to the untreated sample, achieving 64.29% for AFB_1_ and 59.58% for OTA. However, TPA outperformed TPB, indicating that acid treatment is more effective than alkali treatment in enhancing adsorption capacity.

Desorption studies provided insight into the binding strength of the adsorbed toxins at neutral pH. TPA exhibited the lowest desorption values for both AFB_1_ (6.69%) and OTA (15.24%), indicating strong retention of the adsorbed toxins. TPB displayed moderate desorption, with 7.93% for AFB_1_ and 16.77% for OTA, suggesting weaker toxin binding compared to TPA. In contrast, TP showed the highest desorption, with 10.01% for AFB_1_ and 19.19% for OTA, confirming its relatively poor adsorption strength and toxin retention.

These findings suggest that the adsorbent materials have the potential to absorb mycotoxins in the stomach of monogastric animals (low-pH environment) and retain them as they pass through the small intestine. This could inhibit the intestinal absorption of mycotoxins, thereby preserving the integrity of the intestines [31,32].

### 2.7. Kinetics of Adsorption Analysis

The adsorption kinetics of AFB1 and OT onto TP, TPA, and TPB were evaluated using both pseudo-first-order and pseudo-second-order models. The results, shown in Table 2, indicate that the pseudo-second-order model better fits the experimental data compared to the pseudo-first-order model, as evidenced by the higher R^2^ values for both AFB_1_ and OTA adsorption.

For the pseudo-first-order model, the equilibrium adsorption capacities (q_e_) for AFB_1_ ranged from 0.98 mg/g (TP) to 1.20 mg/g (TPA), while for OTA, they ranged from 0.90 mg/g (TP) to 1.08 mg/g (TPA). The R^2^ values for this model were relatively low, with a maximum of 0.6291 for AFB_1_ on TP and 0.5548 for OTA on TPB, indicating a poor fit to the adsorption data. In contrast, the pseudo-second-order model demonstrated significantly higher R^2^ values (greater than 0.99 for all cases), indicating that the adsorption of both AFB_1_ and OTA is more accurately described by this model [33,34]. The equilibrium adsorption capacities (q_e_) for AFB_1_ ranged from 2.74 mg/g (TPA) to 5.29 mg/g (TP), while for OTA, they ranged from 2.65 mg/g (TPA) to 4.88 mg/g (TP). The rate constants (k_2_) also varied, with the highest values observed for TP in both AFB_1_ and OTA (0.08 g/mg/min).

The adsorption of mycotoxins onto three adsorbents is primarily influenced by the interactions between the mycotoxins and the adsorbents. The chemisorption process includes the sharing or exchange of electrons (valency forces) between hydrophilic sites and the divalent ions found on both the adsorbent and the mycotoxin molecules. These interactions enhance the binding affinity, resulting in efficient adsorption [35].

### 2.8. Adsorption Isotherms

Adsorption isotherms were employed to explain the equilibrium relationship between the mycotoxin concentration in solution and the amount adsorbed onto the algal biomass (Table 3). The experimental data were fitted to both Langmuir and Freundlich isotherm models to describe the adsorption behavior.

The Langmuir isotherm assumes monolayer adsorption onto a homogeneous surface. The maximum adsorption capacity (q_m_) obtained from the Langmuir model represents the maximum amount of adsorbate that can be adsorbed per unit mass of adsorbent when the adsorbent surface is completely covered [36].

The Langmuir model fits the adsorption of AFB_1_ on TP well, with a high correlation coefficient (R^2^ = 0.9925) and a maximum adsorption capacity (q_m_) of 0.39 mg/g. However, for TPA and TPB, the Langmuir fit showed lower correlation coefficients (R^2^ = 0.6990 and 0.5279, respectively), indicating a weaker fit. The maximum adsorption capacities of AFB_1_ on TPA and TPB were 1.65 mg/g and 1.64 mg/g, respectively, showing significant improvement in adsorption capacity with chemical treatment, despite the lower fit. For OTA, the Langmuir model also showed a strong fit for TP (R^2^ = 0.9638), with a maximum adsorption capacity (q_m_) of 0.37 mg/g. Both TPA and TPB exhibited improved adsorption capacities of 0.81 mg/g and 0.71 mg/g, respectively, although the correlation coefficient for TPA (R^2^ = 0.9374) was slightly lower than that for TPB (R^2^ = 0.9870).

The Freundlich isotherm describes adsorption on a heterogeneous surface [36]. The Freundlich adsorption isotherm constants, K_f_ and n, were determined for all adsorbent-mycotoxin systems. The K_f_ values reflect the adsorption capacity, while the n values indicate the adsorption intensity [36,37].

The Freundlich isotherm provided a better overall fit for both AFB_1_ and OTA adsorption on all adsorbents. The correlation coefficients (R^2^) for AFB_1_ adsorption on TP, TPA, and TPB were 0.9929, 0.9987, and 0.9982, respectively, indicating a strong fit for the Freundlich model. The Freundlich constant (K_f_) values were 13.39 mg/g for TP, 10.87 mg/g for TPA, and 10.94 mg/g for TPB, showing good adsorption capacity across all samples. The values of n (1.13 for TP, 1.04 for TPA, and 1.04 for TPB) suggest a favorable adsorption process across all adsorbents.

Similarly, for OTA adsorption, the Freundlich isotherm showed excellent fit with R^2^ values of 0.9843 for TP, 0.9936 for TPA, and 0.9956 for TPB. The K_f_ values were 14.61 mg/g for TP, 12.74 mg/g for TPA, and 13.28 mg/g for TPB, indicating higher adsorption capacity for OTA than AFB_1_. The n values for OTA adsorption (1.16 for TP, 1.11 for TPA, and 1.12 for TPB) suggest that the adsorption process was favorable under all conditions.

Overall, whereas the Langmuir model indicated significant improvements in adsorption capacity for chemically treated samples, the Freundlich isotherm provided a better fit across all adsorbents, suggesting a heterogeneous adsorption process for both AFB1 and OTA on TP, TPA, and TPB. Based on the literature review, the simultaneous adsorption of AFB_1_ and OTA on various adsorbents, such as chitosan and bentonites, has been shown to follow the Freundlich isotherm model [38,39]

## 3. Materials and Methods

### 3.1. Chemical and Reagents

Citric acid monohydrate, sulfuric acid, and sodium hydroxide were supplied by Merck (Darmstadt, Germany). HPLC-grade methanol, and acetonitrile were obtained from Macron (Haryana, India). All chemicals used were of analytical grade. A Milli-Q system (Millipore, Molsheim, France) was used as a source of deionized water. Mycotoxin standards: aflatoxin B_1_ (AFB_1_) and ochratoxin A (OTA) were obtained from Fermantek, Jerusalem, Israel. From the solid standards, individual stock solutions were prepared at a 1 mg/mL concentration in an appropriate solvent and stored based on the manufacturer’s instructions. All standard solutions were kept in amber glass vials at −20 °C and brought to room temperature before use. The stock solutions were renewed every 2 months. Polytetrafluoroethylene (PTFE) filters and LC glass vials were purchased from Vertical (Vertical Chromatography Co. Ltd., Nonthaburi, Thailand).

### 3.2. Algal-Based Adsorbents

*Spirogyra* sp. biomass was collected as a byproduct of the bioactive compound extraction process from *Spirogyra* sp. The algal pulp was dried at 60 °C for 24 h in a hot air oven and subsequently ground to a particle size of less than 500 μm using a mechanical grinder. Particle size distribution was analyzed using a laser particle size analyzer. The particles were stored for future use as an adsorbent (denoted as TP).

### 3.3. Chemical Treatment

Ten grams of algal powder (TP) were mixed with 100 mL of 98% sulfuric acid or 1.0 M sodium hydroxide solution and subjected to 120 min of mixing, followed by 30 min of sonication. The treated samples were washed with deionized water until neutral pH was achieved, dried at 105 °C for 6 h, and ground to a particle size of less than 100 μm. The acid-treated algal powder sample was denoted as TPA and the alkali-treated algal powder sample was denoted as TPB.

### 3.4. Adsorbent Characterization

The characteristics of the developed adsorbents were thoroughly analyzed utilizing various types of characterization methods. The surface morphology of the adsorbents was examined using scanning electron microscopy (SEM: Quanta 450 SEM, FEI, Hillsboro, OR, USA) and operating at a voltage of 10 kV. The samples were stuck to stubs using double-sided tape and then covered with a coating of gold before evaluation. The specific surface area and porosity were evaluated using nitrogen adsorption utilizing the Brunauer–Emmet–Teller (BET) method (3Flex Micromeritics Instruments, Norcross, GA, USA) at liquid nitrogen temperature (−196 °C). Before testing, each sample was vacuum-pretreated at 350 °C for 16 h. The pore size distribution was examined according to the Barrett–Joyner–Halenda (BJH) method based on the adsorption isotherm. Furthermore, functional groups were identified using Fourier Transform Infrared Spectroscopy (FTIR: PerkinElmer Spectrum100, PerkinElmer, Shelton, CT, USA). The samples were finely ground, and potassium bromide (KBr) pellets were prepared for the infrared analysis. The study included a range of wave numbers from 4000 to 400 cm^−1^. This method determines chemical functional groups by detecting certain molecular vibrations.

### 3.5. Batch Adsorption Studies

Adsorption studies were conducted to investigate the adsorption efficiency of a prepared adsorbent for AFB_1_ and OTA. The adsorbent was placed in Eppendorf tubes and subjected to controlled conditions in an incubator shaker, which maintained a constant temperature of 37 °C and speed up to 250 rpm. Key parameters such as contact time, adsorbent dosage, pH, and toxin concentrations were varied to assess their effects on adsorption percentage.

Adsorption experiments involved adding 1.00 mL of the mycotoxin solution at different pH levels to the adsorbent. After reaching equilibrium, the samples were centrifuged at 10,000 rpm for 30 min, and the supernatant was analyzed for residual toxin concentrations using HPLC (Water 2475 Multi Fluorescence Detector, Water Corp., Milford, MA, USA) after appropriate dilution and filtration. The separation utilized a C18 analysis column (Inertsil^®^ ODS-3, with dimensions of 150 × 4.6 mm and a particle size of 5 µm; GL sciences Inc, Japan). The AFB_1_ detection was performed using a mobile phase of water, methanol, and acetonitrile in a 50:40:10 (*v*/*v*/*v*) ratio, and a detection wavelength of 365 nm. The OTA detection was performed using a mobile phase of 6% acetic acid in water and acetonitrile at a ratio of 50:50 (*v*/*v*), with specific excitation and emission wavelengths set at 333 nm and 443 nm, respectively.

Various factors influencing adsorption were systematically studied. The effect of contact time was evaluated by sampling at intervals from 15 to 120 min, using an adsorbent concentration of 1.0% *w*/*v* at pH 3, with initial toxin concentrations of 1.0 mg/L for AFB_1_ and OTA. The impact of adsorbent dosage was tested by varying the dosage from 0.001% to 5.0% *w*/*v*, while maintaining constant stirring speed, pH, contact time, and volume. The influence of pH was examined across a range of 3 to 9, using 0.1 M citric acid and 0.2 M sodium phosphate for pH adjustment, and maintaining an adsorbent dosage of 0.5% *w*/*v* and a constant temperature (37 °C). Additionally, the effect of toxin concentration was assessed by varying concentrations from 0.02 to 2.0 mg/L, under fixed conditions of adsorbent dosage, pH, temperature, and stirring speed. The adsorption capacity (mg/g) (Equation (1)) was calculated using the following equation [40]:

Adsorption capacity (q_e_; mg/g) = [(C_o_ − C_e_)/m] × V (1)

where C_o_ and C_e_ are the initial and equilibrium mycotoxin concentrations, respectively, V is the volume of solution used, and m is the adsorbent dosage.

### 3.6. Adsorption Kinetics

Adsorption kinetics indicate the correlation between the adsorption process and time, showing the principles of adsorption rate and dynamic equilibrium. Investigating adsorption kinetics indicates mass transfer actions and the influence of material properties on adsorption, and provides insights into the possible adsorption mechanisms at operation [34]. In this study of mycotoxin adsorption kinetics, pseudo-first-order and pseudo-second-order kinetic models were used to describe the adsorption rate of mycotoxins onto the surface of adsorbent [33,34].

The pseudo-first-order kinetic model (Equation (2)) indicates that adsorption is determined by the diffusion process and can be described as follows:

ln(q_e_ − q_t_) = lnq_e_ − k_1_t (2)

The pseudo-second-order model (Equation (3)) indicates that the adsorption rate is determined by a chemisorption mechanism, characterized by electron sharing or transfer between the adsorbent and the adsorbate. The model can be expressed as follows:

t/q_t_ = (1/k_2_q_e_^2^) + t/q_e_ (3)

where q_e_ (mg/g) and q_t_ (mg/g) are the amounts of mycotoxin adsorbed in mg/g of the adsorbent at equilibrium and at time t (min), respectively. Additionally, k_1_ is the first-order adsorption rate constant (1/min), and k_2_ is the second-order adsorption rate constant (g/mg/min).

### 3.7. Adsorption Isotherm Models

The adsorption behavior of mycotoxins on untreated and chemically treated algal biomass was evaluated using Langmuir (Equation (4)) and Freundlich (Equation (5)) isotherms to describe the adsorption equilibrium [41,42].

The Langmuir model assumes monolayer adsorption on a homogeneous surface. The linear form of the Langmuir isotherm is given by

C_e_/q_e_ = (1/q_m_K_L_) + (C_e_/q_m_) (4)

The Freundlich model is an empirical equation suitable for describing adsorption on heterogeneous surfaces. The linear form of the Freundlich isotherm is

ln q_e_ = ln K_F_ + (ln C_e_/n) (5)

where C_e_ (mg/L) is the equilibrium concentration of the adsorbate in solution, q_e_ (mg/g) represents the equilibrium adsorption capacity, and q_m_ (mg/g) indicates the maximum adsorption capacity. K_L_ and K_F_ represent the Langmuir isotherm constant and the Freundlich isotherm constant, respectively. Additionally, n denotes the coefficient of adsorption strength.

## 4. Conclusions

This study demonstrates the significant potential of modified *Spirogyra* sp. algal biomass as an efficient adsorbent for removing the mycotoxins AFB_1_ and OTA from aqueous solutions. The findings highlight that various factors, including contact time, adsorbent dosage, pH, and initial mycotoxin concentration, play crucial roles in determining adsorption performance.

Optimal adsorption was achieved at a contact time of 60 min, with the TPA sample exhibiting removal efficiencies of 91% for AFB_1_ and 68% for OTA. Increasing adsorbent dosage improved adsorption efficiency, though excessive dosages led to particle aggregation, reducing the effective surface area. The optimal dosage of 5.0 mg/mL balanced adsorption efficiency with minimal adsorbent usage. Acidic to neutral pH conditions (pH 5–6) were found to favor adsorption, particularly for TPA, by enhancing functional group accessibility and binding interactions with the mycotoxins. Across all pH values tested, TPA outperformed other algal biomass powders, demonstrating a higher affinity for both AFB_1_ and OTA.

Kinetic studies revealed that the adsorption of both mycotoxins followed a pseudo-second-order model, indicating that the process is governed by second-order kinetics. However, TPB showed a higher adsorption capacity for AFB_1_ (q_e_ = 4.03 mg/g) compared to TPA (q_e_ = 2.74 mg/g), underscoring the efficacy of alkali treatment in improving adsorption characteristics. Isotherm modeling showed that the Freundlich isotherm model best described the adsorption of both mycotoxins, indicating a heterogeneous adsorption process, while the Langmuir model provided a good fit for AFB_1_ on TPA and TPB, suggesting some degree of surface homogeneity.

Overall, the modified *Spirogyra* sp. biomass is very effective at adsorbing both AFB_1_ and OTA. The results illustrate the significance of algal biomass treatment methods in enhancing mycotoxin adsorption and reducing risks related to mycotoxin contamination in food and feed products. Future research should investigate the long-term stability of modified algal biomass and its effectiveness in practical applications for enhanced food safety measures.

## Figures and Tables

**Table 1:** Surface area and pore size of adsorbent.

Adsorbent	Surface Area (m^2^/g)	Pore Size (Å)
TP	6.39	75.93
TPA	6.77	49.73
TPB	7.51	69.98

algal powder (TP); acid−treated algal powder (TPA); alkali−treated algal powder (TPB).

**Table 2:** The adsorption kinetics parameters of AFB_1_ and OTA onto TP, TPA, and TPB.

Kinetic	Parameters	AFB_1_			OTA		
		TP	TPA	TPB	TP	TPA	TPB
Pseudo- first-order	q_e_ (mg/g)	0.98	1.20	1.05	0.90	1.08	1.02
	k_1_ (1/min)	0.001	0.001	0.0003	0.001	0.001	0.001
	R^2^	0.6291	0.5242	0.2435	0.4827	0.4882	0.5548
Pseudo-second-order	q_e_ (mg/g)	5.29	2.74	4.03	4.88	2.65	3.14
	k_2_ (g/mg/min)	0.08	0.04	0.07	0.08	0.04	0.05
	R^2^	0.9993	0.9982	0.9987	0.9972	0.9972	0.9937

algal powder (TP); acid−treated algal powder (TPA); alkali−treated algal powder (TPB); aflatoxin B_1_ (AFB_1_) and ochratoxin A (OTA).

**Table 3:** The Langmuir and Freundlich isotherm parameters for the adsorption of AFB_1_ and OTA onto TP, TPA, and TPB.

Isotherm	Parameters	AFB_1_			OTA		
		TP	TPA	TPB	TP	TPA	TPB
Langmuir	q_m_ (mg/g)	0.39	1.65	1.64	0.37	0.81	0.71
	K_L_	0.42	0.10	0.10	0.64	0.33	0.36
	R^2^	0.9925	0.6990	0.5279	0.9638	0.9374	0.9870
Freundlich	K_f_ (mg/g)	13.39	10.87	10.94	14.61	12.74	13.28
	n	1.13	1.04	1.04	1.16	1.11	1.12
	R^2^	0.9929	0.9987	0.9982	0.9843	0.9936	0.9956

algal powder (TP); acid−treated algal powder (TPA); alkali−treated algal powder (TPB); aflatoxin B_1_ (AFB_1_) and ochratoxin A (OTA).

## Data Availability

Data is contained within the article.
